# A Cross-Cultural Comparison of Dietary Intake in University Students from the United Arab Emirates and the United Kingdom

**DOI:** 10.3390/nu17193094

**Published:** 2025-09-29

**Authors:** Sarah Dalibalta, Yara Elmashak, Aseel Amer, Yousef Abusaker, Andrea McNeilly, Gareth W. Davison

**Affiliations:** 1 College of Arts and Sciences, American University of Sharjah, University City, Sharjah 26666, United Arab Emiratesaamer@aus.edu (A.A.);; 2 Sport and Exercise Sciences Research Institute, Ulster University, Belfast BT15 1ED, UK; a.mcneilly@ulster.ac.uk (A.M.); gw.davison@ulster.ac.uk (G.W.D.)

**Keywords:** dietary habits, nutrition, cross-cultural analysis, UK, UAE

## Abstract

**Background/Objectives**: The occurrence of non-communicable diseases (NCDs) globally is rising rapidly, largely due to modifiable risk factors such as unhealthy diets. Studies have shown that poor dietary habits are prevalent among university students and may persist in later life, increasing the risk of chronic health conditions. The objective of this study was to evaluate the diet of two different groups of university students, in the United Arab Emirates (UAE) and United Kingdom (UK), with the aim of identifying areas for intervention to improve overall health and wellbeing. **Methods**: Detailed 7-day diet diaries were collected from undergraduate university participants in the UAE and UK. Diet diaries were quantitatively assessed using Nutritics software generating reports on mean intakes for energy, macro- and micronutrients. Independent sample *t*-tests were utilized to compare nutrient intake between cohorts in the two different regions. **Results**: A total of 158 students participated in this study. Results showed significant differences in intake levels in most macronutrients and micronutrients (*p* ≤ 0.05). Upon comparison, UK participants consumed diets higher in sugar (+9.4 g/day), saturated fat (+4.2 g/day), cholesterol (+90 mg/day), and sodium (+307 mg/day) compared to their UAE counterparts, placing them at risk of cardiovascular diseases (CVDs). Cholesterol intake was oversufficient in both UAE and UK males by 40% and 57%, respectively. In UAE females, there were notable deficiencies in protein intake, omega 3, vitamin D, iron, iodine, and folic acid (*p* ≤ 0.05), placing them at risk of CVDs, anemia, diabetes, and cancer. Interestingly, both UAE males and females were 100% deficient in dietary vitamin D intake. **Conclusions**: Nutritional imbalances should be addressed through campus-based nutrition education programs. This study also highlights the importance of dietary guidelines targeted at specific populations accounting for cultural differences.

## 1. Introduction

A balanced and healthy diet is important for development and growth and is vital in the prevention of many diseases, including non-communicable diseases (NCDs) [1]. NCDs are chronic diseases which include CVDs, stroke, cancer, chronic respiratory diseases, and diabetes [1]. These conditions are widespread, and account for 74% of all deaths globally [1]. The development of NCDs can be attributed to five main risk factors: unhealthy diets, physical inactivity, excessive alcohol consumption, smoking and air pollution [1]. These risk factors are largely preventable, and early interventions can have a high impact. One of the key modifiable risk factors associated with NCDs, and a principal common causal factor for many of its conditions is undoubtedly an unhealthy diet. This is a major contributor to overweight and obesity and is associated with a greater risk of early onset CVD and type-2 diabetes [2]. It is estimated that worldwide adult obesity has doubled since 1990 whilst adolescent obesity has quadrupled [2]. The prevalence of overweight and obesity among children and adolescents aged 5–19 has risen from 8% in 1990 to 20% in 2022 [2]. The well-established link between unhealthy diets and disease illustrates the need for much research on dietary intakes especially in countries that are strongly affected by NCDs.

The United Arab Emirates (UAE) ranks among the top countries with a high percentage of individuals suffering from NCDs. The mortality rate in the UAE associated with NCDs is 77%, with the main risk factors including unhealthy diets, high body mass index, high systolic blood pressure, high fasting plasma glucose, and high total cholesterol [3]. Hence, the UAE national agenda has committed to reducing NCD deaths by promoting a healthy lifestyle and spreading health awareness especially among youth. On the other hand, in Europe, NCDs are responsible for 90% of annual deaths [4], with the same behavioral risk factors responsible for over 85% of the NCD burden. More specifically in England, the mortality rate attributable to NCDs has been reported as 88.8% in 2019, with high body mass index, smoking and high fasting plasma glucose as the top three risk factors for morbidity and mortality [5].

Young adults (18–25 years old) in transition from adolescence to adulthood are particularly vulnerable to weight gain as their diet and lifestyle changes. Therefore, understanding the diet habits of adolescents and young adults is vital as good habits are built early and are beneficial in avoiding the risk of disease later in life. Many reports from North American universities have showed significant weight gains between 1 and 4 kg in university students, coining the term “Freshman 15”, in reference to the gain of up to 6.8 kg during their first year of university [6]. A meta-analysis from 2015 found that more than 61% of students gained weight with an average of 3.4 kg over the first academic year [6]. Although reports from the UK are not as extensive, a longitudinal weight change was evaluated across 23 universities in England and similarly found a mean gain of 3.46 kg in over 51% of the sample, with 75% of students undergoing a meaningful weight change in their first year [7]. In a mixed methods study in Scotland covering 1313 young adults, overweight/obesity prevalence was found to be at 22%; only 40% reported eating an adequate number of fruits and vegetables and 60% had more than four unhealthy snacks a day [8]. Fewer reports from the UAE exist but a recent survey of 4728 female university students showed changes in food preferences and an increase in the consumption of processed foods, sugary drinks, and fast food, highly correlated with overweight and obesity [9]. Another study from Zayed University students in the UAE showed that knowledge and healthy dietary habits were both lacking [10].

As poor life habits gained during university may persist throughout adulthood and predispose individuals to higher risk of disease, notably NCDs, this study aimed to ascertain dietary patterns in young university students in the UAE. Since most of the current research to date determines dietary patterns mainly through a survey or questionnaire approach, this work is relatively unique, particularly in the UAE, as it aims to achieve a more comprehensive quantitative assessment of food consumption patterns and nutrient intake in university students. Moreover, we were keen to compare food and nutrient intake in university students of the same age group in a different geographical population, such as in the UK, where the NCD risk is also very high. This could inform public health initiatives to develop dietary guidelines and promote healthier food patterns consistent with specific population and cultural preferences.

## 2. Materials and Methods

### 2.1. Study Population

University students from the American University of Sharjah (AUS) in the United Arab Emirates (UAE) and from Ulster University (UK) volunteered to take part following written informed consent alongside institutional ethical approval (AUS committee reference number IRB 240/296 and Ulster University committee reference number MSc130). Participants were recruited through university-wide announcements and email invitations. Eligibility criteria included healthy students aged between 18 and 24 years with no limit on gender or minority status. Participants consisted of 20 males (19.8%) and 81 females (80.2%) from AUS, and 23 males (40.4%) and 34 females (59.6%) from Ulster University; overall ratio of males to females was 0.46:1.

### 2.2. Study Design

To assess nutritional intake, participants were asked to keep a diet diary of all food and drink consumed for seven consecutive days. Participants recorded all meals and snacks daily. To ensure accurate and detailed data, participants were required to include approximate portion sizes or weight of each entry, the ingredients, and the brands of food items or recipes of composite dishes. All participants were provided with necessary guidance in estimating portion size and on how to measure the weight of food. All participants were instructed to describe the cooking method used (i.e., boiled, roasted, fried, etc.). Participants were also asked to include self-reported height and weight, to allow for the calculation of body mass index (BMI).

### 2.3. Dietary Software Analysis

All dietary analysis was completed using Nutritics software (Nutritics, 2019, v5.09) [11]. This software contains a large database of common food and drinks found in various cuisines, including Middle Eastern. For cases where the food or drink consumed was not available in the database, individual recipes were generated in Nutritics to allow for nutritional analysis. The 7-day food diaries were inputted into the software for analysis, which then generated mean intakes for energy (kcal), and calculated macro- and micro-nutrient intake values, expressed as grams (g) or milligrams (mg) per day as appropriate. Recommended reference intakes for micronutrient minerals and vitamins were also obtained using the Nutritics software using built in Daily Reference Values (DRVs). This software has been validated and used in studies in UK adolescents [12] and adults [13].

### 2.4. Statistical Analysis

To compare nutrient intake between male and female cohorts within the two different regions, an independent-sample *t*-test was utilized using SPSS software (IBM SPSS Statistics, Armonk, NY, USA, v28.0.1.0). Statistical significance was determined at *p* ≤ 0.05, and all differences are denoted by an asterisk sign (*). A similar statistical analysis was also conducted for the intake in grams, mg, or µg of the assessed macro- and micronutrients using *t*-tests to compare between the UK and the UAE. To control for the occurrence of a Type I statistical error, the Bonferroni correction was applied to all between-group analyses. An a priori power analysis was performed (G*Power V 3.1.9.7) based on data from Aidoud et al. (2019) [14], where a significant difference in macronutrient intake between two university populations was observed. Assuming α = 0.05, power = 0.80, and effect size = 0.75 (moderate), it was estimated that a total of 102 participants (51 in each group) would be necessary to detect differences in nutrient intakes between the UAE and the UK.

## 3. Results

### 3.1. Participant Characteristics

A total of 101 university students from the American University of Sharjah (AUS) in the UAE and 57 from Ulster University (UK) agreed to participate in this study. At AUS, 20 males and 81 females volunteered to take part with an average age of 20.2 years old, while 23 males and 34 females volunteered from Ulster University with an average age of 20.5 years. The BMI of all participants was calculated. In the UAE, the mean BMI was 22.6 (24.8 for males and 22.1 for females) and in the UK the mean BMI was 22.3 (22.6 for males and 22.1 for females). Moreover, the average overall daily energy intake was 1535 kcal in the UAE and 1857 kcal in the UK. Overall, participants in both the UK and the UAE were of normal weight according to their mean BMI values with a slightly below average mean total energy requirement per day. To understand the nutrient intakes of each cohort, the average daily intakes of macro- and micronutrients were assessed in detail using the Nutritics software.

### 3.2. Macronutrient Intake

The average daily intake of macronutrients in the UAE and UK sample populations was evaluated and compared to reference recommended dietary allowances. As shown in Table 1, the daily intake of all macronutrients in the UK sample population exceeded that of the UAE participants. UK participants consumed significantly more carbohydrates (+28.1 g/day, 95% Cl: 7.9 to 48.3, *p* < 0.01), protein (+15.7 g/day, 95% Cl: 6.4 to 25, *p* < 0.001) and fat (+8.2 g/day, 95% Cl: 1 to 15.5, *p* < 0.05) compared with the UAE participants. Saturated fat, fiber and cholesterol intakes were consistently higher in the UK compared to the UAE (all *p* < 0.05). No significant differences were observed for sugar and trans-fatty acid intake between groups. This illustrates that the dietary patterns in these countries differ substantially.

**Figure 1:**
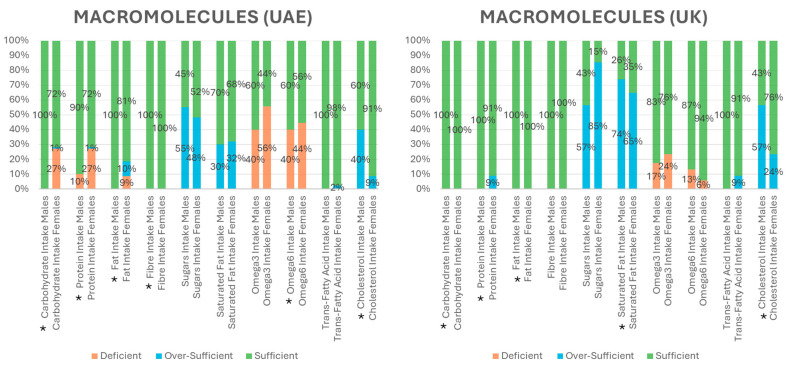
The percent intake of select macronutrients in the UAE and the UK indicating deficient, sufficient and over-sufficient values as per reference dietary allowances. * *p*-value ≤ 0.05 within sample population.

To further analyze the macronutrients that exhibited differences between sample populations, Figure 1 displays deficient, over-sufficient, or sufficient percent intakes, according to references set for each nutrient needed to maintain health. We also observed gender differences in regard to percent intake of select macronutrients within and between sample populations (*p* ≤ 0.05) (Figure 1). For instance, 65% of females and 74% of males in the UK were found to be over-sufficient in intake of saturated fats in contrast to approximately 30% of males and females in the UAE (*p* ≤ 0.05). The average daily intake of cholesterol was also higher in the UK sample, with 57% of males and 24% of females exceeding the recommended daily cholesterol intake. However, 40% of the male participants and approximately 9% of female participants in the UAE were over-sufficient in their daily cholesterol intake. Moreover, males and females in the UK sample population were found to be fully sufficient in their required percent intake of carbohydrates and fats while the UAE sample population contained deficiencies, particularly in females. Another discrepancy between sample populations was related to protein intake. Results showed that nearly the entire UK sample was fully sufficient in protein intake while 10% of males and 27% of females in the UAE population were deficient in protein intake. Both groups of students had oversufficiency in sugar intake, especially UK females. The UAE sample population also contained most deficiencies in omega 3 and 6, with 40% of males and 56% of females in the UAE deficient in omega 3, compared to only 17% of males and 24% of females in the UK. Additionally, 40% of males and 44% of females in the UAE population were omega 6 deficient, whereas only 13% males and less than 6% females in the UK population were deficient. Figure 1 also shows statistical differences between males and females within each sample population. In the UAE, only fiber intake was statistically higher in males than females (*p* ≤ 0.05), whereas in the UK, carbohydrate, protein, fat, saturated fat and cholesterol intakes were all statistically higher in males than females (*p* ≤ 0.05).

### 3.3. Mineral Intake

The average daily intake of minerals was assessed, to include sodium, potassium, chloride, calcium, phosphorus, magnesium, iron, zinc, copper, and manganese, all of which are reported in milligrams (mg) as shown in Table 2. Selenium and iodine are measured in micrograms (µg). Analysis of micronutrient intake demonstrated that UK participants consumed higher levels of several minerals and trace elements. Sodium intake was higher in the UK than in the UAE (+307 mg/day, 95% Cl: 70–543, *p* < 0.01). Likewise, potassium (∆ = +769 mg, *p* < 0.001), chloride (∆ = +1390 mg, *p* < 0.001), calcium (∆ = +140 mg, *p* < 0.05), phosphorus (∆ = +259 mg, *p* < 0.01), magnesium (∆ = +63 mg, *p* < 0.001), iron (∆ = +3 mg, *p* < 0.01), zinc (∆ = +2 mg, *p* < 0.01), and copper (∆ = +0.21 mg, *p* < 0.05) were all significantly higher in the UK group. Iodine intake was also markedly higher in the UK (+41 µg, *p* < 0.001). By contrast, manganese and selenium did not differ significantly between groups. In both populations, however, the mineral intake appeared to fall within recommended dietary allowances with the exception of phosphorus that was higher than recommended values in both UK and UAE participants.

**Figure 2:**
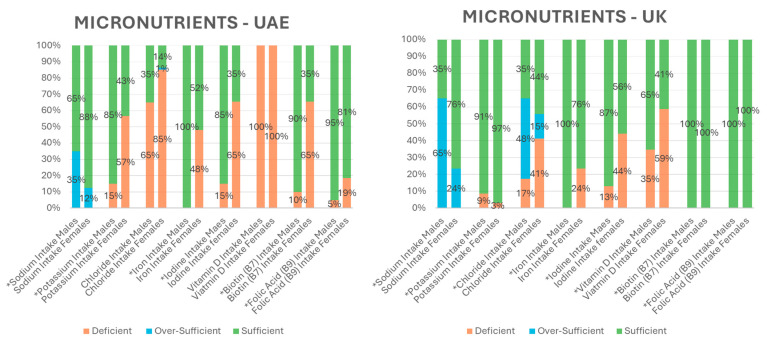
The percent intake of select micronutrients in the UAE and the UK indicating deficient, sufficient and over-sufficient values. * *p*-value ≤ 0.05 within sample population.

Figure 2 displays a comparison of select micronutrients demonstrating sizeable variations between sample populations. The intake of sodium was particularly high in the UK population, with over 65% of males and 24% of females over-sufficient. In the UAE, 35% of males were over-sufficient in sodium, compared to only 12% of females. Moreover, across both the UAE and UK, all males met the requirements for daily iron intake. However, 24% of females in the UK and 48% in the UAE were deficient in their intake of iron. The intake of potassium also varied greatly between the two populations. About 9% of males and 3% of females were potassium-deficient in the UK population; however, almost 57% of females and 15% of males were potassium-deficient in the UAE. Another mineral of interest is iodine which exhibited a high level of deficiency in both the UAE and UK populations, particularly for females. About 65% of females in the UAE were deficient in iodine compared to 44% of females in the UK. Chloride intake also differed significantly between sample populations as 17% of males and 41% of females in the UK were deficient in chloride but 48% and 15% were over-sufficient, respectively. Conversely, in the UAE, only 1% of females were over-sufficient in their chloride intake but more than half (65% of males and 85% of females) were deficient. Both sample populations showed significant differences within each cohort between males and females in almost all minerals studied (*p* ≤ 0.05). Particularly in the UAE population, mineral intake was higher in males than females.

### 3.4. Vitamin Intake

The intake of vitamins in both populations was assessed and represented in Table 3, including vitamin A, vitamin D, vitamin K1, vitamin B12, and biotin (B7) in micrograms (µg), and vitamin E, thiamin (B1), riboflavin (B2), niacin (B3), panthothenic acid (B5), vitamin B6, and vitamin C as milligrams (mg). Apart from vitamins K1 and C, comparisons between the UAE and UK sample populations exhibited significant differences in the intake of all assessed vitamins. Mean intakes were consistently higher in the UK, with delta changes ranging from modest to substantial. For instance, the mean intake of vitamin A was significantly higher in the UK vs. the UAE cohort (+409 µg, *p* < 0.01), as were vitamin D (+1.84 µg, *p* < 0.01), vitamin E (+2.5 mg, *p* < 0.01) and folic acid (+73.5 µg, *p* < 0.01) dietary intakes. Moreover, vitamins B3 and B6 exceeded recommended dietary intakes in both cohorts, while vitamin K1 was below recommended intake in both.

Figure 2 also displays select vitamins, namely vitamins D, B7, and folic acid (B9) as they exhibited the largest variation in percent intake. A major distinction was between the daily intake of vitamin D from the diet. In the UK population, approximately 35% of males and 59% females were deficient in vitamin D intake (Figure 2). Yet, in the UAE population, 100% of males and females were vitamin D dietary deficient. Another significant difference between populations was the level of biotin or vitamin B7 intake (*p* < 0.05). A total of 100% of the UK cohort were fully sufficient in biotin; however, there were deficiencies in this vitamin in the UAE population, particularly in females, whereby 10% of males and over 65% of females were biotin-deficient. Similarly, 100% of the UK sample population were fully sufficient in folic acid intake while ~19% of females and 5% of males in the UAE population were deficient. Within each cohort, males had a significantly higher intake of vitamins B7 and B9 in both the UAE and the UK compared to female counterparts, as well as males having higher vitamin D dietary intakes than females in the UK population only (*p* < 0.05).

## 4. Discussion

Unhealthy eating, mainly through the consumption of ultra-processed foods, is a major component of obesity, particularly in low- and middle-income countries [1,2]. Furthermore, a high intake of sodium, saturated fats, and added sugars, may increase the risk of obesity and hypertension, which is an underlying mechanism for the development of NCDs, specifically CVDs and diabetes [24,25,26]. This study highlighted the nutritional intakes of university students in the UAE and in the UK. Those years are crucial, and the majority of students have been found to gain weight due to poor eating habits, stress, lower physical activity, and an environment with greater access to fast food. Since adolescent weight gain is highly linked to overweight and obesity in adulthood [27], we therefore considered it pertinent to study this subpopulation. Table 4 summarizes key macronutrient and micronutrient differences between UK and UAE sample populations in this study. Female and male participant data was combined, and intakes were classified as insufficient, adequate or in excess to generate overall country-level summaries. If more than 50% of participants fell into a given category, that classification was applied to our UK or UAE sample as a whole.

The analysis of the macronutrient intake in participants in the UAE and the UK showed that individuals in the UK tend to have diets that are richer in sugars, saturated fats and cholesterol potentially placing them at a higher risk of developing CVDs in comparison to the UAE population [28,29]. On the other hand, the UAE sample exhibited a high deficiency in protein intake which may lead to altering energy levels and expenditure, increased risk of fatty liver disease and increases in body mass [30]. Furthermore, the UAE population may also be at risk due to deficiencies in omega 3 and 6 in their diets, known to be important for mental health as well as for the clearance of arterial plaques [31,32]. The cross-cultural analysis between the sample populations in regard to the intake of minerals indicated that the UK sample population had a higher intake of sodium, known to raise the risk of hypertension [33,34]. In contrast, high potassium deficiencies were prevalent in the UAE sample population and may lead to GI disturbances as well as possible respiratory and cardiovascular complications [35]. Both female sample populations may be at a higher risk of anemia due to their deficiency in iron intakes [36], as well as in iodine necessary for normal thyroid hormone synthesis [37]. Furthermore, although both cohorts exhibited low levels of chloride, the UAE sample had deficiencies to a greater extent which puts them at a higher risk of developing diabetes since low levels of chloride (<95–105 mEq) have been associated with increased fasting blood glucose levels [38].

Similarly, the analysis of vitamin intake displayed considerable differences between sample populations whereby the UAE sample was found to have significant deficiencies in comparison to the UK sample. Vitamin D intake was significantly lower in the UAE sample as the data showed that 100% of UAE participants failed to meet the required dietary intakes. This finding is noteworthy as vitamin D is vitally important for musculoskeletal and mental health and may be associated with an increased risk of cancer and diabetes [39,40,41]. It is also consistent with reports of vitamin D deficiency in the UAE in various age groups at an alarmingly high 82.5% of the population [42]. Despite abundant sunlight—religious practices, cultural dress codes, limited exposure to the sun due to associations with heat and exhaustion, and darker skin color have restricted cutaneous vitamin D synthesis [42]. These factors combined with low dietary vitamin D intake as shown in this study have contributed to significant vitamin D deficiencies within the UAE population.

Similarly, the UAE sample population showed a significantly greater percentage of vitamin B7 deficiency, linked to the development of hyperglycemia due to a decrease in insulin and an increase in glucagon secretions [43,44]. There were also notable deficiencies in folic acid associated with anemia and a potential decline in brain health [45]. This is of particular importance in females because deficiencies in folic acid during pregnancy can lead to birth defects such as neural tube defects, placental abruption, premature birth, and low birth weight [46]. A study performed in Dubai Hospital in the UAE that included women from 17 nationalities, found that 53% of deliveries where placental abruption occurred belonged to UAE locals [47]. This clinical observation supports our findings in regard to folic acid deficiency in the UAE sample population.

The differences that exist between the two countries relating to each variable are intrinsically associated with existing cultural differences. These differences are broad and varied, and include, but are not limited to the types, amount and timing of foods consumed, as well as amounts of physical activity and sedentary behavior experienced mainly in the UAE. In fact, the UAE has experienced a rapid economic and social transformation over the last few decades that has led to rapid nutritional transitions with decreased physical activity among this more urbanized and wealthy population [48]. This change in diet is characterized by an increased consumption of calorie dense snacks and sweetened beverages, as well as low fruit and vegetable intake and more frequent snacking. Studies in UAE adolescents have reported higher frequencies of out-of-home and fast-food consumption, in addition to frequent meal skipping or late eating habits [49]. Moreover, non-Emirati youth, who may differ in socioeconomic background from their Emirati counterparts, were shown to have somewhat healthier habits [50]. Findings from the National Diet and Nutrition Survey in the UK indicate that most adults do not meet the UK government recommendations for fruit and vegetable intake, and the average consumption of sugar-sweetened soft drinks and saturated fats exceeded recommendations [51]. However, adults in higher income households were closer to meeting some dietary recommendations such as an increased intake of fruits and vegetables, with most eating occasions occurring at home. Hence, studies in the UK point to clear socioeconomic inequalities in relation to specific food consumption [52]. These differences in eating behaviors, concurrent with socioeconomic and sociocultural influences may explain dietary differences observed in the UAE vs. the UK.

## 5. Strengths and Limitations

A limitation of this study is obviously the dependence of results on the participants’ dedication to filling in their diet diaries accurately across all seven days. Although 7-day weighed food records are more accurate than using other methods, such as the 24 h recall or 3-day diet diary, there is a possibility that it is not completely representative of the individuals’ dietary habits as it is based on self-reporting, which is vulnerable to underreporting or overreporting. Hence, we recognize potential recall and reporting bias in this study, despite the comprehensive guidance offered to each participant. Moreover, a non-probability sampling method was used, which may result in selection bias. We acknowledge that sampling bias may also limit the generalizability of findings to the overall population and that the different sample sizes and unequal ratio of males to females in this study could be a limitation in the representation of each gender. Moreover, there is a notable gender imbalance between both cohorts, with particularly more females in the UAE cohort, that may reduce generalizability; hence, findings should be interpreted within the context of these specific university populations. There is also potential for systemic cultural differences in reporting practices between students in the UAE and the UK that may influence the accuracy of dietary records. We also observed differences in total energy intakes between groups and that may partly influence observed micronutrient intake, which should be considered when interpreting these findings. While multivariate analyses could further strengthen our findings, the sample size was not sufficient to allow robust modeling. Future studies with larger samples may consider multivariate approaches to better account for age, BMI, and other covariates. Despite these limitations, our findings on nutrient deficiencies and excesses among the youth in the UAE and UK such as deficiencies in dietary intake of iron, iodine, and vitamin D in contrast to high intakes of cholesterol and sodium, particularly in the UK, agree with the available literature on broader regional trends in food consumption. For instance, according to WHO, the MENA region severely suffers from malnutrition due to micronutrient deficiencies, particularly in intakes of iron, iodine, zinc, calcium, folic acid, and vitamin D [53]. These deficiencies are responsible for 35% of the total mortality of the region and 144 million disability-adjusted life years (DALYS) in children below 5 years old as well as increasing risks of obesity, CVD and diabetes [53]. Comparably, dietary trends in Europe show that there is a high prevalence of deficiencies in micronutrients such as vitamin D, potassium, folic acid, calcium, selenium, and iodine [54]. Additionally, Europe is ranked as having the highest prevalence of high cholesterol worldwide as it affects 54% of adults, including 133.3 million adults in Germany, France, Italy, Spain and the UK [55]. Food consumption patterns in Europe also indicate that the average intake of dietary salt in adults is between 7 and 13 g daily which is significantly higher than the recommended daily salt intake of less than 2.3 g/day [56].

## 6. Conclusions

NCDs account for a high percentage of deaths worldwide and the risk factors include obesity, physical inactivity, diet, smoking, hyperglycemia, and cholesterol. Most of these risk factors are highly linked to lifestyle patterns, especially diet. This study aimed to quantitatively assess the dietary habits of university students in the UAE and UK using diet diaries and Nutritics software analysis. To our knowledge, this study is the first to perform a quantitative cross-cultural diet analysis between the UAE and the UK. The results found in this study highlight major nutritional issues in these university student populations with obvious lifestyle and cultural differences that need further investigation. Universities could address the nutritional imbalances observed by implementing campus-based nutrition education programs, and reformulating cafeteria menus to promote healthier options. This could include a reduction of sodium in UK cafeteria meals and an increase in vitamin D fortified foods in UAE student meals. Health authorities should consider policies that enhance iron, iodine, vitamin D, and folic acid, especially in the UAE, due to the substantial deficiencies reported in this study. This can include fortification initiatives, such as mandatory vitamin D fortification of dairy in the UAE, tailored to local needs. Therefore, these findings should aid nutritionists and healthcare professionals in properly advising young people with regard to diet and, as such, decreasing the susceptibility of disease. Findings support the need for culturally tailored dietary guidelines to reduce NCD risk.

## Figures and Tables

**Table 1:** Mean daily macronutrient intake in the UAE and the UK in comparison to reference recommended dietary allowances. * Significant at *p*-value ≤ 0.05 comparing UK vs. UAE.

Macronutrient Intake					
	Mean ± Standard Deviation (UAE)	Mean ± Standard Deviation (UK)	∆ (UK − UAE)	95% Cl	Recommended Dietary Allowance
Carbohydrate Intake (g)	185.2 ± 53.3	213.3 ± 66.8 *	28.1	7.88–48.32	130 g/day [15]
Carbohydrate Intake (kcal)	751.3 ± 222.2	853.2 ± 267.2 *	101.9	20.11–183.69	
Carbohydrate Intake (kcal%)	45.7	44.75	−0.95	NA	
Protein Intake (g)	76.8 ± 28.9	92.5 ± 28.6 *	15.7	6.38–25.02	Males: 67 g/day Females: 57 g/day [16]
Protein Intake (kcal)	307.4 ± 118	370 ± 114.6 *	62.6	24.99–100.21	
Protein Intake (kcal%)	18.4	19.1	0.7	NA	
Fat Intake (g)	65 ± 26.6	73.2 ± 25.55 *	8.2	−0.23–16.63	
Fat Intake (kcal)	585.2 ± 239.5	658.8 ± 229.9 *	73.6	−2.19–149.39	
Fat Intake (kcal%)	35.5	34.3	−1.2	NA	<30% kcal/day [17]
Starch (g)	114.9 ± 33.4	132.6 ± 38.6 *	17.7	6.37–29.03	130 g/day
Fibre (g)	14.65 ± 5.9	20.2 ± 8.0 *	5.6	2.88–8.32	25–30 g/day [18]
Sugars (g)	69.2 ± 28.1	78.6 ± 39.5	9.4	−0.04–18.84	90 g/day [19]
Saturated Fat (g)	21.4 ± 9.5	25.6 ± 10.3 *	4.2	1.36–7.04	Males: <30 g/day Females: <20 g/day [20]
Omega 3 (*n*-3) (g)	0.62 ± 0.57	0.94 ± 0.58 *	0.32	0.13–0.51	Males: 1.6 g/day Females: 1 g/day [21]
Omega 6 (*n*-6) (g)	3.46 ± 3.04	5.01 ± 2.5 *	1.55	0.67–2.43	Males: 6.4 g/day Females: 5 g/day [21]
Trans-Fatty Acid (g)	0.95 ± 0.68	0.99 ± 0.54	0.04	−0.15–0.23	<2.2 g/day [22]
Cholesterol (mg)	248.2 ± 129.5	338.2 ± 200.7 *	90	32.1–147.9	<300 mg/day [23]

**Table 2:** The average daily mineral intake in the UAE and the UK in comparison to reference recommended dietary allowances for males and females. * Significant at *p*-value ≤ 0.05 comparing UK vs. UAE.

	Daily Mineral Intake					
	Mean ± Standard Deviation (UAE)	Mean ± Standard Deviation (UK)	∆ (UK − UAE)	95% Cl	Recommended Dietary Allowance for Males	Recommended Dietary Allowance for Females
Sodium (mg)	1967 ± 762	2274 ± 708 *	307	70.63–543.37	>575 < 2400	>500 < 2300
Potassium (mg)	2272 ± 751	3041 ± 1005 *	769	469.79–1068.21	>2000	>1600
Chloride (mg)	1717 ± 816	3107 ± 987 *	1390	1088.37–1691.63	>2300 < 3600	>2300 < 3600
Calcium (mg)	764 ± 318	904 ± 368 *	140	26.1–253.9	>400–450	>400
Phosphorus (mg)	1172 ± 453	1431 ± 464 *	259	109.62–408.38	700	700
Magnesium (mg)	242 ± 90	305 ± 121 *	63	27.02–98.98	>190	>150–190
Iron (mg)	10 ± 3	13 ± 4 *	3	1.81–4.19	>4.7	>8
Zinc (mg)	8 ± 3	10 ± 3 *	2	1.03–2.97	>5.5	>4
Copper (mg)	1.05 ± 0.52	1.26 ± 0.60 *	0.21	0.02–0.4	>1.0 < 1.6	>0.4
Manganese (mg)	4.87 ± 6	4.95 ± 5	0.08	−1.67–1.83	>1.4	>1.4
Selenium (µg)	61 ± 24	59 ± 24	−2	−9.79–5.79	>40	>20 < 400
Iodine (µg)	92 ± 59	133 ± 73 *	41	18.83–63.17	>70	>70

**Table 3:** The average daily vitamin intake in the UAE and the UK in comparison to reference recommended dietary allowances for males and females. * Significant at *p*-value ≤ 0.05 comparing UK vs. UAE.

	Vitamin Intake					
	Mean ± Standard Deviation (UAE)	Mean ± Standard Deviation (UK)	∆ (UK − UAE)	95% Cl	Recommended Dietary Allowance for Males	Recommended Dietary Allowance for Females
Vitamin A (µg)	562 ± 563	971 ± 790 *	409	176–642	>300	>400 < 7500
Vitamin D (µg)	2.12 ±1.34	3.96 ± 2.53 *	1.84	1.13–2.55	>2.5 < 80	>2.5 < 80
Vitamin E (mg)	6.6 ±3.1	9.1 ± 4.1 *	2.5	1.28–3.72	>4	>3 < 540
Vitamin K1 (µg)	45.6 ± 44.4	37.7 ± 44.7	−7.9	−22.3–6.58	120	90
Thiamin (B1) (mg)	1.27 ± 0.41	1.82 ± 0.63 *	0.55	0.37–0.73	>0.23	>0.5 < 100
Riboflavin (B2) (mg)	1.48 ± 0.56	1.96 ± 0.81 *	0.48	0.24–0.72	>0.8	>0.8 < 40
Niacin (B3) (mg)	25.2 ± 11.2	40.6 ± 13.7 *	15.4	11.23–19.57	16	14
Pantothenic Acid (B5) (mg)	4.8 ± 2	6.7 ± 2.5 *	1.9	1.14–2.66	>3 < 7	>3 < 7
Vitamin B6 (mg)	1.55 ± 0.56	1.96 ± 0.62 *	0.41	0.22–0.6	>1.2 < 1.7	>1.4
Folic Acid (B9) (µg)	208 ± 95.6	281.5 ± 110.1 *	73.5	39.37–107.63	>100	>100 < 1000
Vitamin B12 (µg)	4.61 ± 3.3	6.38 ± 3.8 *	1.77	0.59–2.95	>1	>1 < 2000
Biotin (B7) (µg)	23.7 ± 15.1	36.75 ± 18.3 *	13.05	7.46–18.64	>10 < 200	>25 < 60
Vitamin C (mg)	77.3 ± 63.7	95.2 ± 59.5	17.9	−4.01–39.81	>10	>40 < 2000

**Table 4:** Summary of key macronutrient and micronutrient differences between UK and UAE sample populations in this study. Overall status was generated from male and female data combined for each country. Excess (≥50% participants exceed recommended intake values-oversufficient), adequate (≥50% participants fall within recommended intake values- sufficient), and insufficient (≥50% participants are below recommended intake values-deficient). If <50% then classified by category with the largest proportion of participants.

Nutrient	Mean (UK vs. UAE)	Key Difference (∆ = UK − UAE)	UK Status (Overall)	UAE Status (Overall)
Sugars (g)	78.6 vs. 69.2	UK higher (+9.4)	Excess	Excess
Saturated Fat (g)	25.6 vs. 21.4	UK higher (+4.2)	Excess	Adequate
Cholesterol (mg)	338.2 vs. 248.2	UK higher (+90)	Adequate	Adequate
Sodium (mg)	2274 vs. 1967	UK higher (+307)	Excess	Adequate
Chloride (mg)	3107 vs. 1717	UK higher (+1390)	Adequate	Insufficient
Potassium (mg)	3041 vs. 2272	UK higher (+769)	Adequate	Adequate
Iron (mg)	13 vs. 10	UK higher (+3)	Adequate	Adequate
Iodine (µg)	133 vs. 92	UK higher (+41)	Adequate	Adequate
Vitamin D (µg)	3.96 vs. 2.14	UK higher (+1.84)	Adequate	Insufficient
Folic Acid (B9) (µg)	281.5 vs. 208	UK higher (+73.5)	Adequate	Adequate

## Data Availability

The data presented in this study is available on request from the corresponding author. The data is not publicly available due to privacy reasons.

## Abbreviations

The following abbreviations are used in this manuscript:

NCD	Non-communicable disease
CVD	Cardiovascular disease
BMI	Body mass index
UAE	United Arab Emirates
WHO	World health organization
UK	United Kingdom
